# Timing of first antenatal care visits and number of items of antenatal care contents received and associated factors in Ethiopia: multilevel mixed effects analysis

**DOI:** 10.1186/s12978-021-01275-9

**Published:** 2021-11-17

**Authors:** Berhanu Teshome Woldeamanuel, Tadesse Ayele Belachew

**Affiliations:** Salale University, Fitche, Oromiya Ethiopia

**Keywords:** Antenatal care, Contents of antenatal care, Timing of antenatal care, Schwangerenvorsorge, Inhalte der Schwangerenvorsorge, Zeitplan der Schwangerenvorsorge

## Abstract

**Background:**

Receiving quality antenatal care (ANC) from skilled providers is essential to ensure the critical health circumstances of a pregnant woman and her child**.** Thus, this study attempted to assess which risk factors are significantly associated with the timing of antenatal care and the number of items of antenatal care content received from skilled providers in recent pregnancies among mothers in Ethiopia.

**Methods:**

The data was extracted from the Ethiopian Demographic and Health Survey 2016. A total of 6645 mothers were included in the analysis. Multilevel mixed-effects logistic regression analysis and multilevel mixed Negative binomial models were fitted to find the factors associated with the timing and items of the content of ANC services. The 95% Confidence Interval of Odds Ratio/Incidence Rate Ratio, excluding one, was reported as significant.

**Results:**

About 20% of the mothers initiated ANC within the first trimester, and only 53% received at least four items of antenatal care content. Being rural residents (IRR = 0.82; 95%CI: 0.75–0.90), wanting no more children (IRR = 0.87; 95%CI: 0.79–0.96), and the husband being the sole decision maker of health care (IRR = 0.88; 95%CI: 0.81–0.96), were associated with reduced items of ANC content received. Further, birth order of six or more (IRR = 0.74; 95%CI: 0.56–0.96), rural residence (IRR = 0.0.41; 95%CI: 0.34–0.51), and wanting no more children (IRR = 0.61; 95%CI: 0.48–0.77) were associated with delayed antenatal care utilization.

**Conclusions:**

Rural residences, the poorest household wealth status, no education level of mothers or partners, unexposed to mass media, unwanted pregnancy, mothers without decision-making power, and considerable distance to the nearest health facility have a significant impact on delaying the timing of ANC visits and reducing the number of items of ANC received in Ethiopia. Mothers should start an antenatal care visit early to ensure that a mother receives all of the necessary components of ANC treatment during her pregnancy.

**Supplementary Information:**

The online version contains supplementary material available at 10.1186/s12978-021-01275-9.

## Plain language summary

The third Sustainable Development Goals prioritizes maternal mortality reduction, intending to lower the worldwide maternal mortality rate to 70 per 100,000 live births by 2030. Regular antenatal care from a skilled provider reduces maternal mortality by 20%. The overall quality of ANC service is determined collectively by the timing of ANC, and the contents of ANC received. Though there is an increase in ANC visits and the quality of services received, only 74% of women who gave birth in 2019 received antenatal care from a skilled provider, ranging from 85% in the urban to 70% in the rural. Thus, the quality and content of care might remain poor while the coverage of ANC visits is high. Therefore, it is necessary to analyze the levels and risk factors that affect the timing of ANC visits and contents to assess the quality of ANC services. This is the focus of the current study's research. In this study, nationally representative data from the 2016 Ethiopian Demographic and Health Survey was employed. Our study shows that rural residences, the poorest wealth quintile, no education level, unexposed to mass media, unwanted pregnancy, without decision-making power, and being far from the nearest health facility were found to be factors that hinder early initiation of ANC visits and reduce the number of items of ANC received. In conclusion, we ought to initiate an ANC visit early for a frequent antenatal care visit so that a mother will receive the necessary ANC components.

**Supplementary Information:**

The online version contains supplementary material available at 10.1186/s12978-021-01275-9.

## Abstract (de)

**Hintergrund:**

Eine qualitativ hochwertige Schwangerenvorsorge (ANC) durch qualifizierte Anbieter ist für die Sicherung der kritischen Gesundheitslage einer schwangeren Frau und ihres Kindes unerlässlich. In dieser Studie wurde daher untersucht, welche Risikofaktoren bei Müttern in Äthiopien in signifikantem Zusammenhang mit dem Zeitpunkt der Schwangerenvorsorge und der Anzahl der Inhalte der Schwangerenvorsorge stehen, die in den letzten Schwangerschaften von qualifizierten Anbietern durchgeführt wurden.

**Methoden:**

Die Daten wurden aus dem Ethiopian Demographic and Health Survey 2016 extrahiert. Insgesamt wurden 6645 Mütter in die Analyse einbezogen. Es wurden mehrstufige logistische Regressionsanalysen mit gemischten Effekten und mehrstufige gemischte negative Binomialmodelle verwendet, um die Faktoren zu ermitteln, die mit dem Zeitpunkt und den Inhalten der ANC-Leistungen in Verbindung stehen. Das 95%ige Konfidenzintervall der Odds Ratio/Inzidenzrate, mit Ausnahme von einem, wurde als signifikant angegeben.

**Ergebnisse:**

Etwa 20% der Mütter begannen die ANC innerhalb des ersten Trimesters, und nur 53% erhielten mindestens vier Elemente der Schwangerenvorsorge. Die Tatsache, dass die Mütter auf dem Land wohnten (IRR = 0,82; 95%CI: 0,75–0,90), keine weiteren Kinder wollten (IRR = 0,87; 95%CI: 0,79–0,96) und der Ehemann der alleinige Entscheidungsträger für die Gesundheitsfürsorge war (IRR = 0,88; 95%CI: 0,81–0,96), war mit einer geringeren Anzahl an erhaltenen ANC-Inhalten verbunden. Außerdem waren die Reihenfolge der Geburten von sechs oder mehr (IRR = 0,74; 95%CI: 0,56–0,96), der Wohnsitz auf dem Land (IRR = 0,0,41; 95%CI: 0,34–0,51) und der Wunsch, keine weiteren Kinder zu bekommen (IRR = 0,61; 95%CI: 0,48–0,77) mit einer verzögerten Inanspruchnahme der Schwangerenvorsorge verbunden.

**Schlussfolgerungen:**

Ländliche Wohnorte, der geringste Wohlstand des Haushalts, kein Bildungsniveau der Mütter oder Partner, keine Exposition gegenüber Massenmedien, ungewollte Schwangerschaft, Mütter ohne Entscheidungsbefugnis und eine große Entfernung zur nächsten Gesundheitseinrichtung haben in Äthiopien einen signifikanten Einfluss auf die Verzögerung von ANC-Besuchen und die Verringerung der Anzahl der erhaltenen ANC-Posten. Die Mütter sollten frühzeitig mit der Schwangerenvorsorge beginnen, um sicherzustellen, dass sie während ihrer Schwangerschaft alle notwendigen Bestandteile der ANC-Behandlung erhalten.

## Introduction

Maternal mortality reduction and enhancements in women’s health care are priorities of the third Sustainable Development Goal (SDGs) aimed to reduce the global maternal mortality ratio (MMR) to 70 per 100,000 live births by 2030 [1]. Between 2000 and 2017, the global maternal mortality rate (MMR) was reduced by 38% [2]. In Ethiopia, despite a 71.8% decline in MMR between 1990 and 2015, 1 in 64 women are at risk of dying from maternal-related causes, which is a big gap compared with MMR of 199 per 100, 000 live births plan 2020 [3]. It shows that more effort is needed to achieve the SGDs after ten years. Regular antenatal care from a skilled provider reduces maternal mortality by 20% [4, 5]. According to the 2019 Ethiopian mini Demographic and Health Survey, 74% of women who gave birth in the five years before the survey received antenatal care (ANC) from a skilled provider, ranging from 85% in urban areas to 70% in rural areas [6]. Further, Ethiopia’s DHS 2016 revealed 75% of pregnant women had their blood pressure measured, 73% had a blood sample taken, 66% had a urine sample taken, and 66% received nutritional counseling during their ANC visits [7].

The use of health facilities is significantly associated with ANC visits, and sufficient ANC involves both the use of services and the sufficiency of the content within the services [8, 9]. The 2016 Ethiopia DHS reports that only 20% of women had their first ANC visits in the first trimester, which calls for more ANC attendance [7]. Furthermore, concerning the type of skilled provider, doctors (5.7%), nurses/midwives (42%), health officers (1.4%), and health extension workers (13.2%) received ANC service.

Previous studies regarding antenatal care in Ethiopia and elsewhere recognized that women’s autonomy [10–12], birth order and the number of children born [13–15], husband’s attitude and support [10, 16], lack of money [17] were the main reasons for lower health care utilization. Some studies reported that the education level of mother or husband/partner [10–13, 15, 17–20], age [10, 11, 14, 19], woman’s occupation [10, 17], place of residence [11–15, 17, 20], place of receiving [15, 19], access to mass media [10–13, 15, 17, 18], wealth quintile [10–15, 17–19], and ANC provider [15] were the most important factors that affected the utilization of antenatal care services. According to the literature, wanted pregnancy [12, 15, 17, 19, 20], a lack of health care services such as a long distance to the health facility [10, 17, 19], health insurance [10], and permission to visit a health facility [17] were significant factors associated with antenatal care utilization and service quality.

The World Health Organization (WHO) recommends the first visit received before 12 weeks of pregnancy and the necessary contents of ANC visits to improve women’s care experience and reduce perinatal mortality [21]. Even though there is an increase in ANC visits and the quality of services received, many women are still not timely initiating the first ANC visit in Ethiopia. As a result, they have not received the critical contents of ANC. Though several studies in the past year in Ethiopia have explicitly examined associated factors of antenatal care utilization and completion of four or more visits during pregnancy [11, 14, 16–19], these studies did not investigate the actual number of components of ANC service a woman has received. Besides, these studies revealed that the contents of ANC visit highly influence the effectiveness of the ANC service. Thus, the quality and content of care might remain poor while the coverage of ANC visits is high. The overall quality of ANC service is determined collectively by the timing of ANC, and the contents of ANC received. Therefore, it is necessary to analyze the levels and risk factors that affect the timing of ANC visits and contents to assess the quality of ANC services. This is the focus of the current study's research.

## Methods

### Study setting, data and population

We used population based, nationally representative data from 2016 Ethiopian Demographic and Health Survey (DHS) [7]. The survey was conducted by the Central Statistical Agency (CSA) in collaboration with the Federal Ministry of Health (FMOH) and the Ethiopian Public Health Institute with technical assistance from ICF International and financial support from USAID, the government of the Netherlands, the World Bank, Irish Aid, and UNFPA from January 18, 2016, to June 27, 2016. The 2007 Ethiopia Population and Housing Census sampling frame with 84,915 enumeration areas (EAs), each EAs covering 181 households, was used. The respondents were selected using a stratified two-stage cluster design, each region stratified into urban and rural areas.

First was selecting 645 clusters (202 urban areas and 443 rural areas) with probability proportional to enumeration area size and independent selection within each stratum. In all the selected EAs, the household listing was done from September to December 2015. At the second stage, 28 households were selected per cluster with an equal probability systematic selection involving eligible women aged 15–49 years. Thus, a sample of 16,650 households and 15,683 women aged 15–49 years was identified with a response rate of 94.6%. Furthermore, details of the survey design and methodology have been reported in the 2016 EDHS [7].

Our analysis was based on the records of 6645 (54.3%) women who have complete information on the number of ANC visits, the timing of their first ANC visits, the contents of their ANC visits, and who gave birth in the five years preceding the survey. The latest deliveries were referred to all women.

### Variables

This study has two response variables: Timing of first ANC visits; binary outcome categorized into 1 if a mother starts her first ANC visits within the first trimester (early initiation, or 12 weeks after the onset of pregnancy) and 0 elsewhere. Second, the contents of ANC received during pregnancy (a discrete outcome measured as the number of items WHO recommended and recognized as the contents of ANC) in Ethiopia received by a mother during pregnancy.

Standard guidelines for ANC in Ethiopia recognize that every pregnant woman should receive ANC from a skilled provider that consists of iron supplements, intestinal parasite drugs, at least two doses of Tetanus Toxoid injections, malaria intermittent preventive treatment in pregnancy, and health education on danger signs and complications during pregnancy; blood pressure measurement; urine tests; blood tests; health education on prevention of mother-to-child transmission of HIV/AIDS and HIV/AIDS counseling, testing, and collection of results. The composite index comprises a simple count of items received from skilled providers during the ANC visits. The variable had a minimum value of zero, indicating that the mother had not taken any items or received ANC. A maximum value of ten indicates that she has received all the nationally recommended and recognized content of the ANC. The important explanatory variables explored from previously available literature [10–20] are presented in Table 1.

**Table 1:** Operational definition and categorization of explanatory variables in the study

S. no	Variable	Description and categories
1	Region	Geographic region of resident of mother: 1 = Tigray, 2 = Affar, 3 = Amhara, 4 = Oromia, 5 = Somali, 6 = Benishangul-gumuz, 7 = South Nations Nationalities and People Region (SNNPR), 8 = Gambela, 9 = Harari, 10 = Dire Dawa, 11 = Addis Ababa
2	Residence	Type of place of residence: 1 = urban 2 = rural
3	Birth order	Birth order of child: 1 = 1st birth, 2 = 2–3, 3 = 4–5, 4 = 6 or above
4	Wealth index	Household wealth index (wealth quintle): 1 = poorest, 2 = 2nd, 3 = 3rd, 4 = 4th, 5 = least poorest or richest
5	Mother education	Highest education level of mother: 1 = no education, 2 = primary, 3 = secondary, 4 = higher
6	Mother age	Age of mother in 5-year groups at time of ANC visits: 1 = 15–19, 2 = 20–24, 3 = 25–29, 4 = 30–34, 5 = 35–39, 6 = 40–44, 7 = 45–49
7	Sex	Sex of household head: 1 = male, 2 = female
8	Mother job	Mother’s occupation: 1 = not working, 2 = agricultural, 3 = sales, 4 = skilled manual, 5 = others*
9	Frequency of reading	Frequency of reading newspaper or magazine: 0 = not at all, 1 = less than once a week, 2 = at least once a week
10	Frequency of listening	Frequency of listening to radio: 0 = not at all, 1 = less than once a week, 2 = at least once a week
11	Frequency of watching	Frequency of watching television: 0 = not at all, 1 = less than once a week, 2 = at least once a week
12	Wanted last pregnancy	Wanted last pregnancy: 1 = wanted then, 2 = Wanted later, 3 = Wanted no more
13	Husband education	Husband/partner's education level: 1 = no education, 2 = primary, 3 = secondary, 4 = higher
14	Decision making	Person who usually decides on respondent's health care: 1 = Respondent alone, 2 = respondent and husband/partner, 3 = husband/partner alone, 4 = someone else/others
15	Problem of getting permission	Problem of getting permission to go in seeking medical care for herself: 1 = big problem, 2 = no problem/Not a big problem
16	Problem in getting money	Problem in getting money for treatment in seeking medical care for herself: 1 = big problem, 2 = no problem/Not a big problem
17	Distance	Problem in distance to health facility in seeking medical care for herself: 1 = big problem, 2 = no problem/Not a big problem
18	Not wanting to go alone	Problem in not wanting to go alone in seeking medical care for herself: 1 = big problem, 2 = no problem/Not a big problem

* Professional/technical/managerial, clerical, unskilled manual, etc

The wealth index was coded as: 1 = poorest, 2 = poorer, 3 = middle, 4 = richer, and 5 = richest. The wealth quintile of women’s households in EDHS is a composite indicator that scores were derived using principal component analysis based on housing characteristics and ownership of household durable goods [7]. National wealth quintiles are compiled by assigning the household score to each usual (de jure) household member, ranking each person in the household population by their score, and dividing the distribution into five equal categories, each comprising 20% of the population.

In EDHS 2016, birth order is a discrete variable ranging from 1 to 20. The proportion of birth orders two versus three and four versus five is nearly equal, so birth orders two and three and four and five were merged for this analysis. Further, the proportion of children with higher birth orders is relatively small, and birth orders of six and higher have been merged since the percentage distribution. The same categories were also used in the earlier study by Muchie [14]. Similarly, the percentage of women working in professional, technical, managerial, clerical, or unskilled manuals after screening for missing variables is too small, and the authors merge these two categories for this analysis. These too-few frequencies may, in turn, affect the parameter estimation.

### Statistical methods of data analysis

Data analysis was done using the “R programming” version 4.0.3. Descriptive statistics of the subjects were summarized using frequency tables. A Chi-square test was performed to observe any association between the timing of the first ANC visit and the independent variables. An F-test based on analysis of variance (ANOVA) was used to examine the mean difference in the numbers of components of ANC received. Furthermore, the multilevel mixed-effects logistic regression was fitted to identify variables associated with the timing of first ANC visits. Meanwhile, multilevel mixed-effects count models were performed to identify factors associated with the number of ANC components received from a skilled provider during pregnancy.

First, a Poisson regression model with a log link was performed [22]. Then fitted Poisson regression checked for the problem of overdispersion (variance can be larger than the mean) or under dispersion (variance can be smaller than the mean) using the likelihood test. It was found that this test was significant. Therefore, the negative binomial (NB) regression model was considered the immediate solution for data analysis [23]. Moreover, the data experiencing excess zeros and overdispersion might be due to these excess zeros. Thus, we performed both the zero-inflated models (Zero Inflated Poisson and Zero Inflated Negative Binomial (ZINB)) and the Hurdle models (Hurdle Poisson (HP) and Hurdle Negative Binomial (HNB)) to check if overdispersion is accounted for due to excess zeros [24].

To account for the correlation between measurements (intra-cluster correlation (ICC)), we used the multilevel mixed-effects models (cluster/region-specific random effects). The use of a multilevel modeling approach accounts for the hierarchical nature of the EDHS data, where households were selected within EA clusters. There could be unobserved characteristics of cluster influencing women’s decision to timely initiate ANC and the number of ANC visits, such as the availability and accessibility of health services, cultural norms, and prevailing health beliefs. The outcomes of households within the same cluster are likely to correlate. Ignoring this correlation can underestimate variability (producing biased standard errors) and present falsely narrow confidence intervals [25–27].

Further, disregarding the hierarchical structure of the data and analyzing it as single-level data leads to incorrect inferences (i.e., high type I errors or loss of power) [28]. Finally, based on the Vuong statistic [29], likelihood ratio test, the Deviance, AIC, and BIC for model comparison, the multilevel mixed-effects negative binomial model best-fit factors associated with the number of items of ANC received from a skilled provider (see Additional file 1). Variables with a 95% confidence interval for the incidence risk ratio (IRR) excluding one were considered statistically significant determinants.

## Result

### Socioeconomic and demographic characteristics of respondents

This study included 6645 women who had given birth within the five years preceding the survey. The background characteristics of women with respect to the timing of ANC visits are given in Table 2. Most women (70.3%) were from rural areas, while only 29.7% were from urban areas. Concerning regions, a slightly higher percentage were from Tigray (14.6%), SNNPR (13.3%), Oromia (11.2%), and Amhara (10.7%), while the smallest percent of women were from Afar (6.1%), Gambela (6.8%), and Harari (6.8%). The median age was 27 years. Around 32% were from the richest, and 23% were from the poorest wealth quintile. The majority (49.5%) of women had no education, 32.8% had primary education, and only 6.6% had a higher education level. Regarding media access, only 2.8%, 16.3%, and 21.2% have read a newspaper or magazine, listened to the radio, and watched television at least once a week during their recent pregnancy.

**Table 2:** Characteristics of mothers and Timing of first ANC visits among mothers (n = 6645), EDHS 2016

Variables	Categories	Number of women		Timing of first ANC visits				p-value^a^
				Late		Within first Trimester		
		n	%	n	%	n	%	
Birth order	1	1248	18.8	593	51.8	551	48.2	< 0.001
	2–3	2064	31.1	925	58.6	654	41.4	
	4–5	1547	23.3	644	64.5	355	35.5	
	6 +	1786	26.9	710	73.7	253	26.3	
Region	Tigray	663	10.0	444	64.9	240	35.1	< 0.001
	Afar	615	9.3	186	65.0	100	35.0	
	Amhara	712	10.7	297	59.4	203	40.6	
	Oromia	964	14.5	377	71.7	149	28.3	
	Somali	749	11.3	237	67.9	112	32.1	
	Benishangul	551	8.3	299	76.9	90	23.1	
	SNNPR	866	13.0	485	77.8	138	22.2	
	Gambela	479	7.2	178	55.8	141	44.2	
	Harari	381	5.7	130	40.9	188	59.1	
	Dire Dawa	340	5.1	104	31.4	227	68.6	
	Addis Ababa	325	4.9	135	37.5	225	62.5	
Type of place of residence	Urban	*1332*	*20.0*	610	43.8	782	56.2	< 0.001
	Rural	*5313*	*80.0*	2262	68.7	1031	31.3	
Wealth index combined/ wealth quintiles	Poorest	*2240*	*33.7*	738	69.7	321	30.3	< 0.001
	2^nd^	*1117*	*16.8*	532	70.4	224	29.6	
	3^rd^	*958*	*14.4*	463	66.3	235	33.7	
	4^th^	*863*	*13.0*	449	67.0	221	33.0	
	Least poor or richest	*1467*	*22.1*	690	45.9	812	54.1	
Highest educational level	No education	*4071*	*61.3*	1588	68.4	733	31.6	< 0.001
	Primary	*1767*	*26.6*	920	59.9	617	40.1	
	Secondary	*520*	*7.8*	252	48.6	267	51.4	
	Higher	*287*	*4.3*	112	36.4	196	63.6	
Age in 5-year groups	15–19	*306*	*4.6*	156	62.9	92	37.1	< 0.001
	20–24	*1374*	*20.7*	595	58.2	428	41.8	
	25–29	*1881*	*28.3*	814	58.7	573	41.3	
	30–34	*1425*	*21.4*	638	63.4	369	36.6	
	35–39	*1058*	*15.9*	453	66.3	230	33.7	
	40–44	*445*	*6.7*	160	63.2	93	36.8	
	45–49	*156*	*2.3*	56	66.7	28	33.3	
	Median age = 27							
Sex of household head	Male	*5436*	*81.8*	2301	62.6	1372	37.4	< 0.001
	Female	*1209*	*18.2*	571	56.4	441	43.6	
Respondent occupation	not working	*3873*	*58.3*	1538	62.3	930	37.7	< 0.001
	Agricultural	*1324*	*19.9*	605	67.1	297	32.9	
	Sales	*729*	*11.0*	354	55.0	290	45.0	
	skilled manual	*220*	*3.3*	117	62.9	69	37.1	
	Others	*499*	*7.5*	258	53.2	227	46.8	
Frequency of reading newspaper or magazine	not at all	*6105*	*91.9*	2615	63.5	1506	36.5	< 0.001
	less than once a week	*410*	*6.2*	193	44.8	238	55.2	
	at least once a week	*130*	*2.0*	64	48.1	69	51.9	
Frequency of listening to radio	not at all	*4938*	*74.3*	2029	64.1	1137	35.9	< 0.001
	less than once a week	*852*	*12.8*	431	57.0	325	43.0	
	at least once a week	*855*	*12.9*	412	54.0	351	46.0	
Frequency of watching television	not at all	*5126*	*77.1*	2182	68.2	1016	31.8	< 0.001
	less than once a week	*568*	*8.5*	268	54.4	225	45.6	
	at least once a week	*951*	*14.3*	422	42.5	572	57.5	
Wanted last child	Wanted then	*5360*	*80.7*	2264	60.4	1483	39.6	0.001
	Wanted later	*888*	*13.4*	429	62.3	260	37.7	
	Wanted no more	*397*	*6.0*	179	71.9	70	28.1	
Husband/partner's education level	no education	*3180*	*47.9*	1101	68.9	498	31.1	< 0.001
	Primary	*2155*	*32.4*	1025	65.7	536	34.3	
	Secondary	*744*	*11.2*	324	51.1	310	48.9	
	Higher	*566*	*8.5*	213	42.9	284	57.1	
Person who usually decides on respondent's health care	respondent alone	*1177*	*17.7*	470	59.0	327	41.0	0.001
	respondent and husband/partner	*4165*	*62.7*	1721	61.1	1096	38.9	
	husband/partner alone	*1287*	*19.4*	484	68.6	222	31.4	
	someone else	*16*	*0.2*	5	62.5	3	37.5	
Problem of getting permission	Big problem	*2056*	*30.9*	763	62.8	452	37.2	0.213
	Not a big problem	*4589*	*69.1*	2109	60.8	1361	39.2	
Problem in getting money	Big problem	*3698*	*55.7*	1537	65.5	810	34.5	< 0.001
	Not a big problem	*2947*	*44.3*	1335	57.1	1003	42.9	
Problem in distance to health facility	Big problem	*3535*	*53.2*	1365	65.9	707	34.1	< 0.001
	Not a big problem	*3110*	*46.8*	1507	57.7	1106	42.3	
Problem in not wanting to go alone	Big problem	*2646*	*39.8*	1037	65.6	543	34.4	< 0.001
	Not a big problem	*3999*	*60.2*	1835	59.1	1270	40.9	
Overall		6645	100	3702	79.9	983	20.1	
				Median months for first ANC visits = 4				

*SNNPR* South Nations Nationalities and Peoples Region

^a^p-values based on Chi-square test for testing proportions difference

On the other hand, concerning decision-making power over women’s health care, about two-thirds (65%) reported that both women and husbands/partners usually decide on respondents’ health care. Further, about 26% said they had a big problem getting permission to seek medical care, 50.1% had a big problem getting money for treatment in seeking medical care, and 44.2% had a far distance to a health facility in seeking medical care. In comparison, 33.7% reported a big problem in not wanting to go alone to seek medical care. In addition, the majority (80%) of women reported that their last child was wanted at the time of pregnancy. In comparison, 14.7% said the pregnancy was wanted later, and 5.3% reported they wanted no more.

### Timing of first ANC visit by some characteristics of women

Only 20.1% of women started their ANC visit within the first trimester, with a median of four months for the first ANC visit. The proportion starting first ANC within the first trimester was lower in the SNNPR (22.2%), Benishangul-gumuz (23.1%), and Somali regions (32.1%), whereas it was higher in Dire Dawa (68.6%) and Addis Ababa (62.5%) cities. More than half (56.2%) of women from urban areas started their first ANC visit within the first trimester compared to 31.1% of rural women. Women who had higher levels of education (63.6%) and primary education (40%) started first ANC within the first trimester compared to uneducated women (31%). Further, women whose husbands/partners had higher education had the highest proportion (57%) of their first ANC visit within the first trimester.

The time to early initiate the first ANC visit was almost uniform among women’s age and occupation. The majority of women who read newspapers or magazines at least once a week (52%), who listen to the radio at least once a week (46%), and who watch television at least once a week (58%) started their ANC visits for their recent pregnancy within the first trimester. On the other hand, the proportion of women who began their first ANC visit within the first trimester increases with women’s autonomy concerning decisions about health care. Most women whose pregnancy was wanted (40%) started their first ANC visit within the first trimester and wanted no more children (25%). Moreover, 43% of women who had no problem getting money, 42.3% of women with a short distance to the nearest health facility, and 41% who had no difficulty going alone in seeking medical care had started their first ANC visit within the first trimester.

### The number of items of ANC content received by some characteristics of mothers

**Fig. 1:**
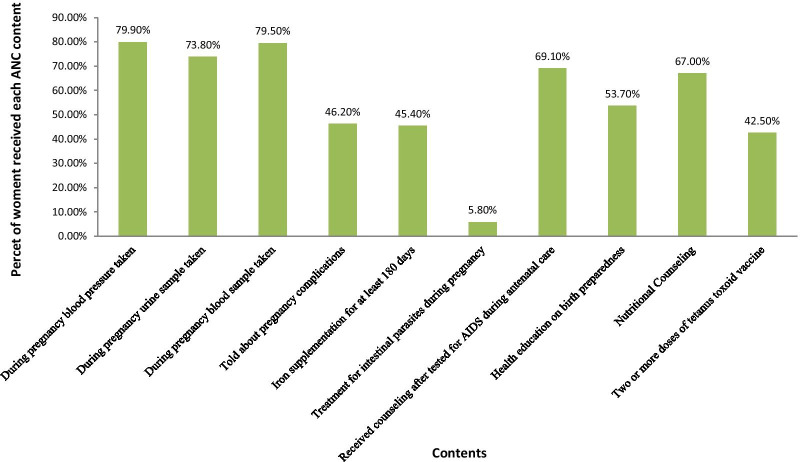
Types of items of ANC Contents received during pregnancy in Ethiopia, EDHS 2016, n = 6645

**Fig. 2:**
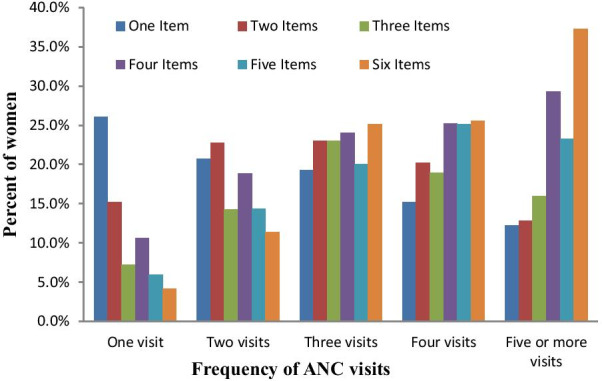
Percentage distribution of number of items of ANC contents received by frequency of ANC visits in Ethiopia, EDHS 2016

Of all women who received ANC at least once, 79.9% had their blood pressure measured, 73.8% had a urine sample taken, and 79.5% had a blood sample taken. Further, 46.3% had been told about pregnancy complications, 45.4% received iron supplementation for at least 180 days, and 5.8% of women received treatment for an intestinal parasite. Additionally, 69.1% received counseling after testing AIDS, 53.7% were informed about birth preparedness, 67% received nutritional counseling, and 42.5% received two or more doses of tetanus toxoid vaccine from a skilled provider during their ANC visits (Fig. 1). The mean number of ANC contents received by a woman was 3.5 items and a standard deviation of 2.2, indicating that the distribution is overdispersed. Figure 2 presents a further detailed examination of the relationship between the frequency of ANC visits and the number of items of ANC contents received. It revealed that the likelihood of receiving the highest number of items of ANC content increases with the frequency of ANC visits. The proportion of women who received six items has monotonically increased from 4.2 to 37.3%, increasing ANC visits from one visit to at least five ANC visits (Fig. 2).

Conversely, the pattern showed a declining trend of the likelihood of receiving only one item or two items, or three items, with an increase in the number of ANC visits. In addition, the timing of the first ANC visit showed a positive association with the mean number of items of ANC contents received. For instance, a woman who started her first ANC visit within the first trimester received, on average, 6.2 items. In comparison, women who had received only one ANC visit had received an average of 3.8 items, compared to virtually six items on average among women with four or more ANC visits (Table 3).

**Table 3:** Item of ANC contents by mother during pregnancy in Ethiopia, EDHS, 2016

Variables	Categories	Mean Number of ANC content	p-value^b^
Birth order	1	6.0	< 0.01
	2–3	5.8	
	4–5	5.3	
	6 +	5.2	
Region	Tigray	6.5	< 0.01
	Afar	4.6	
	Amhara	5.7	
	Oromia	4.7	
	Somali	4.8	
	Benishangul	5.2	
	SNNPR	5.1	
	Gambela	5.2	
	Harari	6.4	
	Dire Dawa	6.1	
	Addis Adaba	7.0	
Place of residence	Urban	6.5	< 0.01
	Rural	5.2	
Wealth index	Poorest	4.7	< 0.01
	2nd	5.1	
	3rd	5.2	
	4th	5.7	
	Richest	6.6	
Highest educational level	No education	5.1	< 0.01
	Primary	5.8	
	Secondary	6.5	
	Higher	6.9	
Age in 5-year groups	15–19	5.2	< 0.01
	20–24	5.7	
	25–29	5.6	
	30–34	5.6	
	35–39	5.5	
	40–44	5.5	
	45–49	5.4	
Sex of household head	Male	5.6	< 0.01
	Female	5.7	
Respondent occupation	Not working	5.5	< 0.01
	Agricultural	5.3	
	Sales	6.0	
	Skilled manual	5.7	
	Others	6.2	
Frequency of reading newspaper or magazine	Not at all	5.4	< 0.01
	Less than once a week	6.9	
	At least once a week	6.9	
Frequency of listening to radio	Not at all	5.3	< 0.01
	Less than once a week	6.1	
	At least once a week	6.3	
Frequency of watching television	Not at all	5.1	< 0.01
	Less than once a week	6.2	
	At least once a week	6.7	
Wanted last child	Wanted then	5.6	< 0.01
	Wanted later	5.7	
	Wanted no more	5.4	
Husband/partner's education level	No education	5.1	< 0.01
	Primary	5.5	
	Secondary	6.2	
	Higher	6.6	
Person who usually decides on respondent's health care	Respondent alone	5.7	< 0.01
	Respondent and partner	5.6	
	Husband alone	5.1	
Problem of getting permission	Big problem	5.0	< 0.01
	Not a big problem	5.8	
Problem in getting money	Big problem	5.2	< 0.01
	Not a big problem	6.0	
Problem in distance to health facility	Big problem	5.1	< 0.01
	Not a big problem	6.0	
Problem in not wanting to go alone	Big problem	5.1	< 0.01
	Not a big problem	5.8	
Timing of first ANC visits	Late	5.2	< 0.01
	Early	6.2	
Frequency of ANC visits	1	3.8	< 0.01
	1–3	4.8	
	4 or more	6.2	
	8 or more	6.9	
Total			

^b^P-values based on ANOVA or F-test for testing the variation among means

### Factors associated with the timing of the first ANC visits: multilevel mixed-effects logistic regression analysis

The multivariable multilevel logistic regression analysis of factors associated with the timing of the first ANC visit is given in Table 4. The likelihood of timely initiating the first ANC visit was lower among six or more birth orders (AOR = 0.74; 95%CI: 0.56–0.96) than the first birth order. Moreover, rural women were 59% less likely to start their first ANC visit within the first trimester (AOR = 0.41; 95%CI: 0.31–0.54) than urban counterparts.

**Table 4:** Mixed effects multilevel analysis determinant of Number of items of ANC contents received, and Timely initiation of antenatal care visits in Ethiopia, EDHS, 2016

Factors	Number of contents of ANC received		Timing of first ANC within first trimester	
	IRR	95% CI	AOR	95% CI
Categories				
Birth order(1st)				
2–3	0.99	0.93, 1.06	0.90	0.73, 1.11
4–5	0.97	0.89, 1.06	0.84	0.66, 1.06
6+	0.96	0.86, 1.06	0.74*	0.56, 0.96
Place of residence (urban)				
Rural	0.82***	0.75, 0.90	0.41***	0.31, 0.54
Wealth index (poorest)				
2nd	1.39***	1.29, 1.49	1.83***	1.54, 2.16
3rd	1.47****	1.37, 1.59	2.02***	1.68, 2.42
4th	1.62***	1.49, 1.75	2.29***	1.87, 2.81
Richest	1.51***	1.36, 1.67	2.17***	1.61, 2.92
Mother education (no education)				
Primary	1.24***	1.17, 1.32	1.73***	1.48, 2.02
Secondary	1.22***	1.10, 1.34	2.14***	1.49, 3.06
Higher	1.21**	1.05, 1.39	5.19***	2.25, 12.03
Mother age (15–19)				
20–24	0.98	0.88, 1.09	0.82	0.61, 1.11
25–29	0.99	0.88, 1.12	0.96	0.69, 1.33
30–34	1.03	0.91, 1.17	1.04	0.74, 1.47
35–39	0.95	0.82, 1.08	0.83	0.57, 1.19
40–44	0.91	0.86, 0.1.16	0.66*	0.44, 0.99
45–49	0.97	0.79, 1.18	0.86	0.53, 1.41
Sex of household head (male)				
Female	0.91***	0.85, 0.97	0.98	0.84, 1.15
Mother occupation (not working)				
Agricultural	1.16	0.09, 1.24	1.03	0.88, 1.20
Sales	1.07	0.99, 1.15	1.11	0.91, 1.37
Skilled manual	1.03	0.91, 1.16	1.05	0.75, 1.47
Others	1.03	0.95, 1.13	1.24	0.94, 1.63
Frequency of reading newspaper/magazine (not at all)				
Less than once a week	0.96	0.88, 1.06	1.08	0.70, 1.65
At least once a week	1.07	0.92, 1.25	1.29	0.59, 2.84
Frequency of listening to radio (not at all)				
Less than once a week	1.12**	1.04, 1.19	1.56***	1.25, 1.93
At least once a week	1.15***	1.07, 1.23	1.49***	1.19, 1.85
Frequency of watching television (not at all)				
Less than once a week	1.09*	1.01, 1.19	1.04	0.81, 1.33
At least once a week	1.04	0.95, 1.14	1.58*	1.11, 2.23
Wanted pregnancy (wanted then)				
Wanted later	0.97	0.91, 1.03	0.84*	0.71, 0.99
Wanted no more	0.87**	0.79, 0.96	0.61***	0.48, 0.77
Decision maker of respondents health care (respondent alone)				
Respondent and partner	0.99	0.76, 1.30	1.21	0.58, 2.53
Husband/partner alone	0.88**	0.81, 0.96	1.19	0.57, 2.49
Someone else/others	0.91	0.69, 1.19	0.76	0.19, 3.03
Problem of getting permission (big problem)				
Not a big problem	1.10***	1.03, 1.17	1.03	0.89, 1.19
Problem in getting money (big problem)				
Not a big problem	0.99	0.95, 1.05	1.04	0.91, 1.20
distance to health facility (big problem)				
Not a big problem	1.19***	1.12, 1.26	1.55***	1.35, 1.78
Husband/partner’s education (no education)				
Primary	1.17***	1.10, 1.24	0.91	0.45, 1.83
Secondary	1.21***	1.11, 1.33	1.29	0.63, 2.61
Higher	1.16***	1.04, 1.30	1.45*	1.08, 1.95
Problem in not wanting to go alone (big problem)				
Not a big problem	1.05	0.99, 1.11	1.06	0.92, 1.23
Number of ANC visits (none)				
1–3	5.12***	4.68, 5.59	–	
4 +	6.08***	5.56, 6.65	–	
Region (Tigray)				
Afar	0.53*	0.45, 0.62	0.17*	0.13, 0.24
Amhara	0.99	0.85, 1.17	0.24*	0.18, 0.32
Oromia	0.68*	0.58, 0.8	0.12*	0.09, 0.16
Somali	0.53*	0.45, 0.62	0.13*	0.1, 0.17
Benishangul-gumuz	0.96	0.81, 1.13	0.3*	0.22, 0.41
SNNPR	0.97	0.83, 1.13	0.26*	0.19, 0.34
Gambella	0.7*	0.59, 0.83	0.15*	0.1, 0.2
Harari	1.03	0.87, 1.22	0.2*	0.14, 0.28
Dire Dawa	1.03	0.87, 1.22	0.63*	0.42, 0.94
Addis Ababa	1.48	1.26, 1.73	.21	0.11, 1.39
cond.(Intercept)	2.29***	1.85, 2.86	1.80	0.99, 3.20

Significant: ‘***’ 0.001 ‘**’ 0.01 ‘*’ 0.05

Reference categories are in parenthesis

The log odds of timely initiating the first ANC visit were higher among the richest wealth quintile (AOR = 2.17; 95%CI: 1.61–2.92), the 4^th^ (AOR = 2.29; 95%CI: 1.87–2.81), and the 3^rd^ (AOR = 2.02; 95%CI: 1.68–2.42) wealth quintile, respectively, as compared to the poorest wealth quintile. The odds of starting the first ANC visit within the first trimester was 5.2 times (AOR = 5.20; 95%CI: 2.25–12.03), 2.14 times (AOR = 2.14; 95%CI: 1.50–3.06) and 1.73 times (AOR = 1.73; 95%CI: 148–2.02), higher among women with a higher, secondary and primary level of education, respectively, as compared to uneducated women after controlling for other variables in the model. Similarly, women whose husbands had higher education levels were 45% (AOR = 1.45; 95%CI: 1.08–1.95) more likely to start their ANC within the first trimester than those whose husbands had not been educated. Women aged 40–44 years old were 34% (AOR = 0.66; 95%CI: 0.44–0.99) less likely to start their first ANC visit on time than women aged 15–19 years old.

Furthermore, women who listened to the radio less than once a week (AOR = 1.56; 95%CI: 1.25–1.93), at least once a week (AOR = 1.49; 95%CI: 1.20–1.85) and watched television at least once a week (AOR = 1.58; 95%CI: 1.11–2.23), respectively, were more likely than those who did not listen to the radio or watch television to start their first ANC in the first trimester. Pregnant women who want no more children were 39% (AOR = 0.61; 95%CI: 0.48–0.77) less likely to start their first ANC visit within the first trimester than those whose pregnancies were wanted. Furthermore, a woman who reported a short distance to a health facility seeking medical care was 55% (AOR = 1.55; 95%CI: 1.35–1.78) more likely to start her first ANC visit within the first trimester (Table 4).

### Factors associated with the number of ANC content items received by a woman: Multilevel mixed-effects Negative binomial analysis

The estimated incidence rate ratio (IRR) indicates that women from rural areas (IRR = 0.82; 95%CI: 0.75–0.90) and female heads (IRR = 0.91; 95%CI: 0.85–0.97) were significantly associated with lower numbers of items of ANC content received. Further, women who wanted no more children (IRR = 0.87; 95%CI: 0.79–0.96), whose husbands/partners decided alone, were significantly associated with lower numbers of items of ANC content received. In contrast, women from the richest wealth quintile (IRR = 1.51; 95%CI: 1.36–1.67), 4^th^ wealth quintile (IRR = 1.62; 95%CI: 1.49–1.75) and 3^rd^ wealth quintile (IRR = 1.47; 95%CI: 1.37–1.59), women who had primary education (IRR = 1.24; 95%CI: 1.17–1.32), secondary education (IRR: 1.22, CI: 1.10–1.34) and higher education (IRR = 1.21; 95%CI: 1.05–1.39) as well as women whose partners had primary education (IRR = 1.17; 95%CI: 1.01–1.24), secondary education (IRR = 1.21; 95%CI: 1.11–1.34) and higher education (IRR = 1.16; 95%CI: 1.04–1.30) were more likely to receive a higher number of items of ANC contents. Additionally, women who have no problem of getting permission (IRR = 1.10; 95%CI: 1.03–1.17), who reported short distance to health facilities (IRR = 1.19; 95%CI: 1.12–1.26), who listen to the radio less than once a week (IRR = 1.12; 95%CI: 1.04–1.19) and at least once a week (IRR = 1.15; 95%CI: 1.07–1.23), who watch television less than once a week (IRR = 1.09; 95%CI: 1.01–1.19), who had received 1–3 ANC (IRR = 5.12; 95%CI: 4.68–5.59) and at least four ANC (IRR = 6.08; 95%CI: 5.56–6.65) from a skilled provider were significantly more likely to have a higher number of items of ANC contents during their pregnancy.

## Discussion

The study found that 53% of women received at least four ANC items, while 20% started their first ANC visit within the first trimester. The multilevel negative binomial regression analysis revealed that the covariates of rural residents and an unwanted child at the time of pregnancy were significantly associated with the lower incidence rate ratio of the number of ANC contents received. Further, the frequency of ANC visits during pregnancy was significantly associated with a higher incidence rate ratio of ANC contents received. In contrast, female heads were significantly associated with a lower incidence rate ratio of ANC contents received. The multilevel logistic regression analysis revealed that having six or more birth orders, living in a rural area, being between the ages of 40 and 44, and wanting no more children were all significantly associated with a lower likelihood of initiating ANC visits on time. Higher wealth quintile, higher education level of women and partners, access to mass media, and a short distance to the health facility in seeking medical care, on the other hand, were significantly associated with increased odds of initiating an ANC visit for a recent pregnancy within the last five years before the survey.

This study showed that higher birth order was inversely associated with the timing of the ANC visit, i.e., women were less likely to start their ANC visit within the first trimester of their sixth or higher birth order. A similar study in Uganda [30] found mothers with third birth orders, compared to those with the first, are about 6–7% less likely to attain the four antenatal visits, and mothers with at least the third birth order are 4–5% less likely to initiate the first visit in the first trimester. Muchie [14], using Ethiopian Mini DHS 2014, also found 38 and 36% lower odds of completing four or more visits of ANC utilization for birth orders of children four or five, and six or more, respectively. One possible reason for this might be mainly in the first pregnancy when women wanted lots of contact with their care provider. Some women would have liked more communication between appointments and were worried about having to deal with pregnancy complications and pain.

Rural mothers are less likely to receive higher ANC content from skilled providers and start ANC visits within the first trimester than urban mothers. This finding is congruent with that of Beeckman et al. [10], who reported higher odds of delaying first ANC visits and ANC visits of less than four among rural mothers. Further, a study done in Bangladesh, [12] found rural mothers are 17% less likely to attend a higher number of ANC visits than urban mothers. Another similar finding from Bangladesh [8] reported that urban mothers were 1.35 times more likely to receive more items of ANC services from a skilled provider than their rural counterparts. In Ethiopia’s rural areas, there is a lack of skilled health care providers, lack of information on antenatal care services, lack of infrastructure, and long distances from health facilities.

Moreover, most mothers in rural Ethiopia were uneducated. Contrary to our findings, Gebremeskel et al. [31] and Weldearegawi et al. [32] reported residence was not associated with the timing of the first ANC visit. This inconsistency might be due to the statistical methodology used and the smaller sample size used by Gebremeskel et al. [31] (n = 409) and Weldearegawi et al. [32] (*n* = 402), whereas the EDHS 2016 used (n = 6645).

Furthermore, we found that women with at least primary education levels are more likely to start the first ANC visit within the first trimester and receive the highest number of items of ANC content from skilled providers. Similarly, women whose partners had at least a primary education were more likely to receive higher ANC content from skilled providers than the uneducated category. Additionally, women whose partners had higher education were more likely to start ANC visits within the first trimester than those without. Further analysis of the 2011 Ethiopian DHS showed that women who had primary education (79%), secondary education (62%), and higher education were 45% times less likely to delay their first ANC visit [10]. Consistent with our finding, Islam [8] also found that there is a 1.12, 1.26, and 1.39 incidence rate ratio of receiving higher numbers of ANC content among women having primary, secondary, and higher education in Bangladesh. But, partners’ primary education level has not significantly increased the incidence of receiving the items of ANC content. In contrast, partners having a secondary or higher education significantly increased the incidence of receiving the items of ANC content [8].

In contrast, in Ghana, Manyeh et al. [33] found no significant effect of husband/partner education level on the timing of ANC visits. Additionally, a systematic review analysis in sub-Saharan Arica found that husband education was significantly associated with uptake, frequency, and timing of first ANC visits [10]. Most likely, this could be because educated women have more access to information and make their own decisions on their health care, which empowered them to exercise, and changed traditional attitudes about using the ANC service. This study suggests that there is an urgent need to focus on mothers’ education. Advocating primary education for girls and encouraging them to pursue secondary or higher education is essential to achieve a tangible change to achieve the sustainable development goals of maternal and infant mortality reduction through effective implementation of maternal health care services [14, 34].

The result also suggests women who listened to the radio and watched television at least once a week were more likely to start their first ANC early and received more items of ANC content from skilled providers. This result agrees with the findings of Yaya et al. [11], where women who watch television at least once a week were 40% less likely to delay their first ANC visit than those who do not watch television at all. But they did not find an association between listening to the radio and the timing of the first antenatal care visit. This variation might be due to a difference in the methodology used. In Bangladesh [8, 12], mass media access was associated with increased ANC content received.

Women whose pregnancies were unwanted or wanted later were more likely to delay their first ANC visit and less likely to receive the highest number of ANC content items than wanted pregnancies. A similar study of the Bangladeshi DHS found that wanted pregnancy was associated with a higher incidence of receiving higher items of ANC contents [8]. Another study from Bangladesh [12], Southern Ethiopia [31], Bahir Dar [35], and Eastern Tigray [32] found unwanted pregnancy significantly associated with delayed initiation of ANC service utilization. This might be that mothers with unwanted pregnancies have anxiety and poor psychological well-being [36] and less attention to pregnancy-related complications, and do not use supplements such as folic acid, vaccinations, health information, and nutritional counseling [37]. Thus, women ought to be encouraged to use modern contraceptives to prevent unwanted pregnancies.

Furthermore, women’s health decision-making power is significantly associated with the content of ANC services received. Women without decision-making power or whose husband/partner alone decides on their health care are strongly associated with lower ANC contents received. This result was congruent with those of northwest Ethiopia [38], Bangladesh [12], the systematic review of sub-Saharan Africa [10], and Tanzania [39]. However, unlike our findings, Gebresilassie et al. [40] found that decision-making on self-care seeking was not significantly associated with the timing of the first ANC visit.

Mothers with a shorter distance to the nearest health facility had better odds of initiating their first ANC visit and receiving items of ANC content from skilled providers. Similar findings are reported in a study in Bahir Dar, Ethiopia [35]. In the Eastern Tigray zone in Northern Ethiopia, distance to the nearest health facility was not a significant predictor of late antenatal care follow-up [32]^.^ In Rwanda, distance to the health facility was not a significant predictor of poor quality of antenatal utilization [41]. Likewise, mothers who had permission to seek medical care were more likely to receive more ANC content.

Lastly, the results indicated that the frequency of ANC visits and timing of the first ANC visit during pregnancy was positively associated with the number of items of ANC contents a woman received from a skilled provider. Women who started antenatal care within the first trimester were more likely to receive more ANC contents items than those who delayed their visit. Likewise, the number of items of ANC content monotonically increases with frequent ANC visits. The findings are consistent with those of [42, 43].

## Conclusions

Findings of this study suggest that rural residences, the poorest wealth quintile, no education level of mothers or partners, unexposed to mass media, unwanted pregnancy, mothers without decision-making power, and a long distance to the nearest health facility have significant impacts on delaying the timing of ANC visits and reducing the number of items of ANC received in Ethiopia. Further, timely initiation of the ANC and the number of ANC visits were significantly associated with the increase in the number of items ANC received during pregnancy. Therefore, this study recommends that women initiate ANC visits timely and frequent antenatal care visits during pregnancy for the quality of ANC received from a skilled provider. Another implication of this study is that educating and empowering girls, particularly in the rural areas, are vital ingredients in all policies aiming to reduce maternal and neonatal deaths through improved quality of antenatal care utilization, particularly in the rural areas. Furthermore, encouraging women to use modern contraceptives, expanding health education in the media, and expanding health facilities are vital inputs that should be included in policies to improve the quality of antennal care utilization, particularly in rural areas. Moreover, women at low economic levels should be given special emphasis.

## Supplementary Information

**Additional file 1: Table S1.** AIC, BIC, Log-likelihood and Deviance based Model comparison for mixed effects models. **Table S2.** Vuong Tests for the non-nested models Poisson, negative-binomial (NB), zero-inflated Poisson (ZIP), hurdle Poisson (HP), zeroinflated NB (ZINB), and hurdle NB (HNB) models. **Table S3.** Akaike’s information criteria (AIC), log-likelihood, and likelihood-ratio for Poisson, negative-binomial regression (NB), mixed Poison (MP) and Mixed NBR (MNBR) models.

## Data Availability

The datasets used and/or analyzed during the current study are available from the corresponding author on reasonable request.

## Abbreviations

ANC: Antenatal care
CI: Confidence interval
CSA: Central Statistics Agency
DHS: Demographic and Health Survey
EDHS: Ethiopia Demographic and Health Survey
EAs: Enumeration areas
FMoH: Federal Ministry of Health
HP: Hurdle Poisson
HNB: Hurdle negative binomial
IRR: Incidence rate ratio
LRT: Likelihood ratio test
MMR: Maternal Mortality Ratio
NB: Negative Binomial
OR: Odds ratio
SDGs: Sustainable Development Goals
UNFPA: United Nations Population Fund
USAID: United States Agency for International Development
WHO: World Health Organization
ZIP: Zero Inflated Poisson
ZINB: Zero Inflated Negative Binomial
