# Supplementary material for: Timing of first antenatal care visits and number of items of antenatal care contents received and associated factors in Ethiopia: multilevel mixed effects analysis
Source: Reprod Health. 2021 Nov 17;18:233. doi: 10.1186/s12978-021-01275-9 (PMC8596955; doi:10.1186/s12978-021-01275-9)
Supplement: Supplementary file 1 — Additional file 1: Table S1. AIC, BIC, Log-likelihood and Deviance based Model comparison for mixed effects models. Table S2. Vuong Tests for the non-nested models Poisson, negative-binomial (NB), zero-inflated Poisson (ZIP), hurdle Poisson (HP), zeroinflated NB (ZINB), and hurdle NB (HNB) models. Table S3. Akaike’s information criteria (AIC), log-likelihood, and likelihood-ratio for Poisson, negative-binomial regression (NB), mixed Poison (MP) and Mixed NBR (MNBR) models. [file 12978_2021_1275_MOESM1_ESM.docx]

Table S1: AIC, BIC, Log-likelihood and Deviance based Model comparison for mixed effects models

| Selection criteria | Models |  |  |  |  |  |
| --- | --- | --- | --- | --- | --- | --- |
|  | M.Poisson | **MNB** | MZIP | MZINB | MHP | MHNB |
| AIC | 28542 | **25723.5** | 29733 | 25726.39 | 25758 | 25761 |
| BIC | 28851 | **26321.9** | 30036 | 26331.68 | 26357 | 26366 |
| Log-likelihood | -14226 | **-12774.8** | -14822 | -12775.2 | -12792 | -12792 |
| Deviance | 28452 | **25549.5** | 29645 | 25550.4 | 25587 | 25585 |

Table S2: Vuong Tests for the non-nested models Poisson, negative-binomial (NB), zero-inflated Poisson (ZIP), hurdle Poisson (HP), zeroinflated NB (ZINB), and hurdle NB (HNB) models.

| Model 1 | Model 2 | **Test Statistic (AIC Corrected)** | p-value | **Better Model** |
| --- | --- | --- | --- | --- |
| Poisson | NB | -14.09035 | < 2.22e-16 | NB |
| Poisson | ZIP | -35.11654 | < 2.22e-16 | ZIP |
| Poisson | HP | -35.09681 | < 2.22e-16 | HP |
| NB | ZINB | -30.66581 | < 2.22e-16 | ZINB |
| NB | HNB | -30.65993 | < 2.22e-16 | HNB |
| ZIP | ZINB | -27.63728 | < 2.22e-16 | ZINB |
| ZIP | HP | 1.390854 | 0.082135 | ZIP/HP |
| ZINB | HNB | 1.390881 | 0.082131 | ZINB |
| HP | HNB | 0.0350458 | 0.48602 | HP/HNB |

Table S3: Akaike’s information criteria (AIC), log-likelihood, and likelihood-ratio for Poisson, negative-binomial regression (NB), mixed Poison (MP) and Mixed NBR (MNBR) models.

| Models | AIC | log-likelihood (df) | LR test (pvalue) |
| --- | --- | --- | --- |
| P | 30584.79 | -15249.4 (43) | 323.67 (<0.0001) |
| MP | 29733.71 | -14822.86 (44) |  |
| NB | 28978.31 | -14445.15 (44) | 320.52 (<0.0001) |
| MNB | 28542.34 | -14226.17 (45) |  |
